# A Phase 1 Study of KHK4083: A Single‐Blind, Randomized, Placebo‐Controlled Single‐Ascending‐Dose Study in Healthy Adults and an Open‐Label Multiple‐Dose Study in Patients With Ulcerative Colitis

**DOI:** 10.1002/cpdd.918

**Published:** 2021-01-29

**Authors:** Kenichi Furihata, Yoh Ishiguro, Naoki Yoshimura, Hiroaki Ito, Shinji Katsushima, Etsuji Kaneko, Munetake Shimabe, Mayumi Mukai, Risa Watanabe, Takuya Morishige

**Affiliations:** ^1^ P‐One Clinic, Keikokai Medical Corporation Tokyo Japan; ^2^ Department of Clinical Research National Hirosaki Hospital, National Hospital Organization Aomori Japan; ^3^ Department of Medicine Division of Gastroenterology Tokyo Yamate Medical Center Tokyo Japan; ^4^ Kinshukai Infusion Clinic Osaka Japan; ^5^ Department of Gastroenterology Kyoto Medical Center, National Hospital Organization Kyoto Japan; ^6^ Kyowa Kirin Co., Ltd Tokyo Japan

**Keywords:** antibody, monoclonal, OX40, pharmacokinetics, phase 1, placebo controlled, safety and tolerability, ulcerative colitis

## Abstract

OX40 plays an essential role in maintaining late T‐cell proliferation and survival by suppressing apoptosis and by inducing T‐cell memory formation. Here, we report the results of the phase 1 study of KHK4083, a fully human antimonoclonal antibody specific for OX40. In this study, we aimed to assess the safety and tolerability of a single intravenous or subcutaneous administration of KHK4083 compared with placebo in healthy Japanese and Caucasian subjects and determined the pharmacokinetics (PK) and immunogenicity. Also, we assessed the preliminary efficacy and pharmacodynamics of multiple intravenous doses in Japanese patients with moderate to severe ulcerative colitis (UC). Drug‐related treatment emergent adverse events occurred in 21 healthy subjects (58.3%) and 5 patients with UC (62.5%) after administration of KHK4083. There were no serious adverse events. The PK profile of a single intravenous dose of 10 mg/kg KHK4083 was similar in healthy Japanese and Caucasian subjects. Of 8 UC patients, a clinical response was observed in 3 patients (37.5%) and clinical remission in 2 patients (25.0%) in week 6. Our study demonstrated the safety and tolerability of single and multiple administrations of KHK4083 in healthy Japanese and Caucasian subjects and Japanese patients with moderate to severe UC. It also indicated favorable pharmacological properties of the drug.

OX40, a member of the tumor necrosis factor (TNF) receptor superfamily, suppresses apoptosis and induces late T‐cell proliferation, survival, and formation of memory T cells.^1^ The OX40 ligand is expressed on antigen‐presenting cells (APCs) at the site of inflammation.^2,^, ^3^ When the ligand is bound to the OX40 receptor, which is expressed on activated T cells, the immune response may be enhanced by mechanisms described above.^4^ The immunopathogenesis of inflammatory diseases such as atopic dermatitis is known to be linked to T‐cell activation by OX40.^5^ Although the mechanism that triggers the development of gastrointestinal inflammatory disorders, including ulcerative colitis (UC), is not well understood, studies suggest that inflammatory bowel diseases in general likely have multifactorial causes such as the imbalance of immune homeostasis (balancing defense and tolerance of microbes) and the involvement of apoptosis of peripheral blood lymphocytes.^6^, ^7^ Consequently, dysregulation of immune response causing constant stimulation and activation of T cells by APCs via cytokine and chemokine production is considered to play a critical role in the pathogenesis of UC.^8^, ^9^

UC is a chronic inflammatory disorder of the colonic mucosa, starting in the rectum and generally extending continuously throughout or in parts of the colon.^10^, ^11^, ^12^ UC is thought to originate from an abnormal epithelial defense system and sustained inflammatory response to commensal and environmental factors in patients with genetic risk factors.^10^ Meta‐analytical data have shown that incidence of UC is high worldwide, up to 24.3 per 100 000 person‐years in Europe, 19.2 per 100 000 person‐years in North America, and 6.3 per 100 000 person‐years in Asia and the Middle East.^13^

The American College of Gastroenterology Clinical Guidelines strongly recommend monoclonal antibodies with anti‐inflammatory properties such as vedolizumab, an antibody specific for alpha4 beta7 integrin (α4β7)^14^ for inducing remission in patients with moderately to severely active UC who have previously failed to respond to anti‐TNF therapy.^15^

KHK4083 is a fully human, nonfucosylated IgG_1_ monoclonal antibody developed by Kyowa Kirin Co., Ltd, which acts in a pharmacologic pathway different from currently available drugs for UC. KHK4083 induces selective depletion of activated T cells by antibody‐dependent cell‐mediated cytotoxicity. It antagonistically suppresses clonal T‐cell expansion by inhibiting the activation of OX40. We expect KHK4083 to be efficacious in treating UC because of its unique pharmacological activity in reducing the number of OX40‐positive lymphocytes. These lymphocytes are effector T cells^3^ and effector memory cells, which may be involved in the chronic inflammatory response and flare of inflammatory bowel diseases.^16^ Because OX40‐positive lymphocytes are often found in colonic biopsies of patients with UC,^16^ we expect KHK4083 will reduce their numbers, thereby alleviating the symptoms of UC. In jejunal and colon biopsy specimens of UC patients, elevated OX40 or OX40 ligand levels were detected along with high α4β7 and MAdCAM‐1 expression, and the number of OX40 positive (OX40^+^) cells was increased in sites of mucosal inflammation.^17^

This phase 1 study was the first to assess the safety and tolerability of single ascending intravenous and subcutaneous administration of KHK4083 in healthy Japanese and Caucasian subjects compared with placebo. We also determined the pharmacokinetics (PK) and immunogenicity of both administrations as well as the preliminary efficacy and pharmacodynamics of multiple intravenous doses of KHK4083 in Japanese patients with moderate to severe UC.

## Materials and Methods

### Study Design

This study consisted of 2 parts: a single‐blind single‐ascending‐dose study of KHK4083 administered to healthy Japanese and Caucasian subjects (part 1) and a multiple‐dose open‐label study of KHK4083 in Japanese patients with UC (part 2).

**Figure 1:**
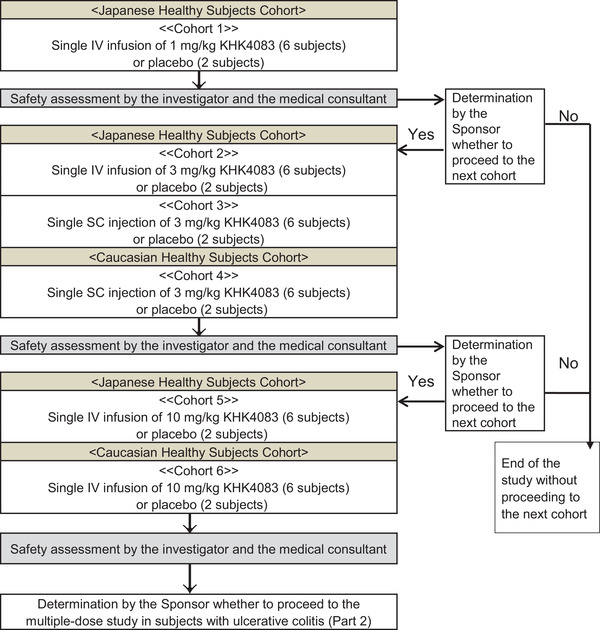
Cohort design of the single‐ascending‐dose study in healthy Japanese or Caucasian subjects (part 1).

In part 1, the study commenced with cohort 1, with a single intravenous infusion of 1 mg/kg KHK4083 (Figure 1 and Figure S1). The dose was increased according to the results of the safety assessment by the investigator and the medical consultant, and subcutaneous injection was introduced. Eight eligible healthy subjects were randomized per cohort to each treatment regimen (KHK4083, 6 subjects; placebo, 2 subjects). Single ascending doses of the investigational product (IP) KHK4083 or placebo were allocated to each subject. For each cohort, safety was assessed up to day 15 or until discontinuation. The sponsor determined whether to increase the dose according to the results of the safety assessment. If the results on day 4 or 5 showed no clinical abnormalities requiring follow‐up and if the investigators determined it to be appropriate after receiving a single dose of IP on day 1, subjects were discharged and returned to the study site for scheduled observations and examinations.

The sponsor determined whether to proceed to part 2 according to the results of the safety assessment in part 1. In part 2, ≥4 patients in cohort 7 with moderate to severe UC were to receive intravenous infusions of 10 mg/kg KHK4083 every 2 weeks for 6 weeks, on days 1, 15, and 29 and followed for an observation period of 16 weeks, on an entirely outpatient basis (Figure S2).

The protocol was approved by the institutional review board (Table S1). The study was conducted in accordance with Good Clinical Practice Guidelines and the Declaration of Helsinki. All subjects provided written informed consent prior to participation.

### Subjects

Subjects enrolled in part 1 were healthy Japanese or Caucasian men ≥ 20 and < 45 years at the time of obtaining the informed consent, with a body mass index of ≥18.5 and <25.0 kg/m^2^ at the screening. Patients enrolled in part 2 were Japanese men or women ≥ 20 years of age at the time of informed consent, with UC diagnosed ≥ 6 months before obtaining the informed consent. Patients were diagnosed with moderate to severe UC with a total Mayo score of ≥4 at screening.^15^ Subjects from both parts had no findings suggestive of tuberculosis, as assessed by chest x‐ray or chest computed tomography at screening, and subjects who had tested negative or below the detection limit for the virus infection test result met all the corresponding criteria.

### Subject Disposition and Demographics

Of the 32 subjects (24 Japanese and 8 Caucasian) administered an intravenous dose, 24 subjects (18 Japanese and 6 Caucasian) received KHK4083 (1‐10 mg/kg), and 8 subjects (6 Japanese and 2 Caucasian) received placebo. Of the 16 subjects (8 Japanese and 8 Caucasian) administered a subcutaneous dose, 12 subjects (6 Japanese and 6 Caucasian) received KHK4083 (3 mg/kg), and 4 subjects (2 Japanese and 2 Caucasian) received placebo. One Caucasian subject who received an intravenous dose of 10 mg/kg KHK4083 (cohort 6) did not show up during follow‐up and withdrew from the study on day 104. All 48 subjects who received the IP (KHK4083, 36 subjects; placebo, 12 subjects) in part 1 and all 8 patients who received KHK4083 in part 2 were included in the safety analysis set and pharmacodynamic analysis set. All 36 subjects who received KHK4083 in part 1 and all 8 patients who received KHK4083 in part 2 were included in the pharmacokinetic analysis set. All 8 patients who received KHK4083 in part 2 were included in the full analysis set (FAS).

In the single‐ascending‐dose study in healthy Japanese and Caucasian subjects, the mean age of each treatment group ranged from 26.2 to 34.1 years (Table 1A, part 1). The mean weight of each treatment group ranged from 65.48 to 68.77 kg in Japanese subjects and from 67.80 to 75.13 kg in Caucasian subjects. Age, weight, and body mass index were similar in the KHK4083 and placebo groups.

**Table 1:** Demographic Characteristics and Pharmacokinetics: (A) Demographic and Other Baseline Characteristics; (B) KHK4083 Pharmacokinetic Parameters

(A) Demographic Characteristics Part I									
		Placebo		KHK4083					
Parameter (Unit)		Japanese n = 8	Caucasian n = 4	1 mg/kg IV n = 6	3 mg/kg IV n = 6	3 mg/kg SC n = 6	3 mg/kg SC (Caucasian) n = 6	10 mg/kg IV n = 6	10 mg/kg IV (Caucasian) n = 6
Age (y)	n	8	4	6	6	6	6	6	6
	Mean	34.1	26.8	33.5	26.2	29.3	31.0	31.5	31.2
	SD	6.6	4.3	8.8	5.6	7.6	6.6	6.8	5.6
	Min	23	24	24	21	23	25	24	26
	Median	36.5	25.0	33.5	24.0	27.5	29.5	30.0	29.5
	Max	41	33	44	35	44	43	41	41
Weight (kg)	n	8	4	6	6	6	6	6	6
	Mean	65.88	71.98	65.58	65.48	68.77	67.80	68.17	75.13
	SD	5.69	9.78	6.80	6.70	5.00	4.14	7.49	5.69
	Min	59.3	63.3	57.7	53.8	61.0	60.1	59.4	66.7
	Median	65.30	70.10	63.95	66.80	68.55	69.45	68.70	76.00
	Max	77.2	84.4	75.9	72.6	76.4	71.2	76.9	83.4
	<70	7 (87.5)	2 (50.0)	4 (66.7)	4 (66.7)	4 (66.7)	3 (50.0)	3 (50.0)	1 (16.7)
	≥70	1 (12.5)	2 (50.0)	2 (33.3)	2 (33.3)	2 (33.3)	3 (50.0)	3 (50.0)	5 (83.3)
BMI (kg/m^2^)	n	8	4	6	6	6	6	6	6
	Mean	22.13	21.93	22.48	21.58	23.00	21.48	22.43	23.62
	SD	1.40	2.38	1.71	1.81	1.58	1.99	1.22	0.94
	Min	19.8	19.6	19.3	18.8	20.7	18.8	21.2	22.1
	Median	22.25	21.85	22.75	21.45	23.50	21.00	21.95	23.60
	Max	24.5	24.4	24.5	24.1	24.4	24.1	24.0	25.0
	<23	6 (75.0)	2 (50.0)	4 (66.7)	5 (83.3)	2 (33.3)	4 (66.7)	4 (66.7)	1 (16.7)
	≥23	2 (25.0)	2 (50.0)	2 (33.3)	1 (16.7)	4 (66.7)	2 (33.3)	2 (33.3)	5 (83.3)

(A) Demographic Characteristics Part 2		
Parameter (Unit)		KHK4083
Age (y)	n	8
	Mean	47.3
	SD	15.7
	Min	23
	Median	50.5
	Max	66
Sex	Female	2 (25.0)
	Male	6 (75.0)
Weight (kg)	n	8
	Mean	68.67
	SD	11.39
	Min	51.1
	Median	70.93
	Max	81.1
	<70	4 (50.0)
	≥70	4 (50.0)
BMI (kg/m^2^)	n	8
	Mean	24.34
	SD	3.64
	Min	18.9
	Median	25.30
	Max	28.9
	<23	2 (25.0)
	≥23	6 (75.0)
Total Mayo score at baseline	n	8
	Mean	5.6
	SD	2.0
	Min	3
	Median	5.5
	Max	9
Partial Mayo score at baseline	n	8
	Mean	4.3
	SD	1.8
	Min	2
	Median	4.5
	Max	7

(B) Single Intravenous or Subcutaneous Dose of KHK4083 in Healthy Japanese or Caucasian Subjects									
Dose	N	n	t_max_(h)	C_max_(μg/mL)	AUC_0‐t_(mg·h/mL)	AUC_0‐∞_(mg·h/mL)	t_1/2_(h)	CL (mL/h/kg)	V_z_ (mL/kg)
1 mg/kg IV (cohort 1)	6	6	1.02 (1.00‐1.08)	21.6 ± 2.7	5.96 ± 970	6.02 ± 1020	239 ± 81	0.170 ± 0.028	56.8 ± 15.2
3 mg/kg IV (cohort 2)	6	6	1.03 (1.00‐1.05)	64.5 ± 8.3	19.9 ± 3300	20.1 ± 3500	263 ± 120	0.153 ± 0.026	54.8 ± 18.8
3 mg/kg SC (cohort 3)	6	6	167.94 (48.00‐167.98)	16.1 ± 3.8	11.7 ± 3600	11.8 ± 3700	178 ± 103	N/A^a^	N/A^a^
3 mg/kg SC (Caucasian) (cohort 4)	6	6	167.96 (96.00‐168.00)	21.1 ± 3.2	16.7 ± 2700	16.9 ± 2900	289 ± 104	N/A^a^	N/A^a^
10 mg/kg IV (cohort 5)	6	6	1.03 (1.00‐1.05)	199 ± 19	79.2 ± 8700	80.5 ± 9500	511 ± 85	0.126 ± 0.015	91.4 ± 9.3
10 mg/kg IV (Caucasian) (cohort 6)	6	6	1.03 (1.02‐1.05)	198 ± 21	83.5 ± 6100	87.9 ± 10 300	547 ± 144	0.115 ± 0.013	88.7 ± 16.8

(B) After the 3rd Dose of KHK4083 Following Intravenous Dose of 10 mg/kg Every 2 Weeks in Japanese UC Patients									
10 mg/kg (UC) (cohort 7)	8	8	1.05 (1.00‐1.15)	297 ± 39	160 ± 36	163 ± 39	440 ± 154	0.0644 ± 0.0138	38.4 ± 7.5

BMI, body mass index; IV, intravenous; Q2W, every 2 weeks; SC, subcutaneous; SD, standard deviation; UC, ulcerative colitis; n, number of subjects used to calculate summary statistics for each parameter.

Percentages are shown in parentheses. t_max_ is shown as median (min‐max) and other pharmacokinetic parameters are shown as mean ± standard deviation.

The pharmacokinetic parameters in parentheses are calculated in the case of SC administration.

^a^Absolute bioavailability of KHK4083 was estimated to be 58.7% based on the mean AUC_0‐∞_ for subcutaneous and intravenous doses of 3 mg/kg in Japanese subjects.

In the multiple‐dose study of UC patients, 6 of 8 patients who received KHK4083 (75.0%) were male. The mean ± standard deviation (SD) for age was 47.3 ± 15.7 years, for weight was 68.67 ± 11.39 kg (4 patients weighed less than 70 kg), and total Mayo score and partial Mayo score at baseline were 5.6 ± 2.0 and 4.3 ± 1.8, respectively (Table 1A, part 2).

### End Points

The primary objective in part 1 was the safety and tolerability of a single intravenous or subcutaneous dose of KHK4083 in healthy Japanese and Caucasian subjects assessed in a randomized, placebo‐controlled, single‐blind comparative study design. The primary objective in part 2 was the safety and tolerability of intravenous doses of KHK4083 administered every 2 weeks in patients with UC, evaluated in an open‐label study design. The secondary objectives were PK and immunogenicity of a single intravenous or subcutaneous dose of KHK4083 in healthy Japanese and Caucasian subjects and multiple intravenous doses of KHK4083, administered every 2 weeks, in Japanese patients with UC.

For the safety analysis, treatment‐emergent adverse events (TEAEs), clinical laboratory tests, vital signs, and 12‐lead electrocardiogram were assessed during the study. All adverse events based on the verbatim terms reported by the investigators were mapped to the lowest‐level term, preferred term, and system organ class of the Medical Dictionary for Regulatory Activities (version 20.1). The TEAEs were defined as any adverse event that had occurred or worsened after IP administration and used for analyses. The safety analysis set consisted of subjects who received 1 or more doses of the IP.

Serum KHK4083 concentration‐time profiles were determined, and PK parameters were assessed. For immunogenicity, the number and percentage of samples that were positive for the anti‐KHK4083 antibody and neutralizing antibody (NAb) were calculated. The serum concentration of KHK4083 and anti‐KHK4083 antibody or NAb was measured using the validated electrochemiluminescent (ECL) method.

Serum concentrations of KHK4083 were measured using an ECL immunoassay.^18^, ^19^ Briefly, the assay consists of an ECL immunoassay in which capture (KM4472, anti‐KHK4083 idiotypic antibody) and detection (ruthenylated KM4473, ruthenylated another anti‐KHK4083 idiotypic antibody) reagents are step‐wise incubated with standards, QCs and samples to form a complex on the assay plate. The KHK4083 in standards, QCs, controls and samples is captured onto the Meso Scale Discovery (MSD) high‐bind Plate coated with the capture reagent. Following the incubation of the detection reagent, MSD read buffer is added and the plate is then read using a MSD ECL plate reader. The ECL signal generated is relative to the amount of KHK4083 present in the standards, quality controls (QCs), controls, and samples tested. The concentration of KHK4083 is calculated off the nonlinear regression of the standards.

The quantification range of the assay was 25.0 to 10 000 ng/mL. In the validation study, the intra‐ and interbatch accuracy, expressed as percentage relative error (%RE), ranged from ‐9.42% to ‐2.14% for the QCs in the quantification range. The precision for QCs, expressed as coefficient of variation (%CV), was ≤4.34% for intrabatch and ≤10.4% for interbatch precision.

Antidrug antibodies (ADAs) were measured by an ECL immunoassay. The serum samples were first purified by solid‐phase extraction and acid dissociation treatment using biotinylated KHK4083. The eluted ADAs were then captured on an MSD standard plate and detected using ruthenylated KHK4083 as the detection reagent. The ECL signal per sample corresponded to the amount of ruthenylated KHK4083, which in turn corresponds to the amount of ADAs bound to the plate.

The procedure consists of 2 assays: screening assay and confirmatory assay. The screening assay is designed to qualitatively detect the presence of ADAs. The samples that are determined to be antibody positive in the screening assay undergo the confirmatory assay. The confirmatory assay evaluates the specificity of the antibodies when samples are incubated with an excess of added free KHK4083. The sensitivity of the ADA assay was 24.623 ng/mL of the positive control (monkey polyclonal antibody) in pooled drug‐naive human serum. The drug tolerance limit of the assay was 100 μg/mL of KHK4083 at 125 ng/mL of the positive control. The intra‐ and interassay precision was ≤19.5% for the screening assay and ≤4.49% for the confirmatory assay.

Confirmed ADA‐positive samples were also analyzed in a neutralizing antidrug antibody (NAb) ECL immunoassay to detect any NAbs. The serum samples are purified through solid‐phase extraction with acid dissociation treatment after incubation with biotinylated KHK4083. The eluted antibodies are then incubated with ruthenylated KHK4083, and a complex is formed between the antibodies and the ruthenylated KHK4083. The mixture is then added to an MSD standard plate coated with OX40 (drug target), and nonneutralized ruthenylated KHK4083 becomes bound to the plate. The ECL signal inversely corresponds to the amount of NAbs present in the samples.

The sensitivity of the NAb assay was 134.977 ng/mL of positive control (anti‐idiotypic antibody) in pooled drug‐naive human serum. The drug tolerance limit of the assay was 25.0 μg/mL of KHK4083 at 250 ng/mL of positive control. Intra‐ and interassay precision of the method was ≤13.6%.

In part 2, cell‐surface markers OX40, CD45, CD4, and CD3 were measured using flowcytometry^20^, ^21^ CD45^+^CD3^+^CD4^+^OX40^+^ lymphocytes were measured as OX40‐positive helper T cells (OX40^+^ Th). Counts of OX40^+^ Th that were not bound to KHK4083 (unoccupied OX40^+^ Th) and total OX40^+^ Th counts were determined using KHK4083‐competitive and KHK4083‐noncompetitive anti‐OX40 antibody. The pharmacodynamic analysis set consisted of patients who followed the treatment regimen and who were available for a postdosing observation.

In part 2, exploratory end points were evaluated from the results of clinical responses that were rated according to the total Mayo score and the partial Mayo score. The Mayo score is one of the most commonly used scoring indexes in studies involving UC.^22^ Composed of 4 categories (bleeding, stool frequency, physician assessment, and endoscopic appearance) rated from 0 to 3, the total score gives a range from 0 to 12. Study has reported that a score of <2.5 on the full index is an optimal score as the patient‐defined remission.^23^ The total Mayo score, obtained at screening and in week 6, was calculated from the results of stool frequency subscore, rectal bleeding subscore, endoscopy subscore, and the Physician's Global Assessment (PGA) score. The partial Mayo score, obtained at screening, every 2 weeks from week 0 up to and every 4 weeks beyond week 6, was calculated from the stool frequency subscore, rectal bleeding subscore, and PGA score.^24^ The clinical response was defined as a decrease of ≥3 from baseline and a ≥30% reduction from baseline in the total Mayo score, with a decrease in the rectal bleeding subscore of ≥1 or a rectal bleeding subscore ≤ 1. Clinical remission was defined as a total Mayo score ≤ 2, with no individual subscore > 1. The FAS for the efficacy analysis excluded UC patients who had not been administered the IP and those without postdosing efficacy data.

## Results

### Pharmacokinetics

#### Part 1: Single‐Ascending‐Dose Study in Healthy Japanese and Caucasian Subjects

**Figure 2:**
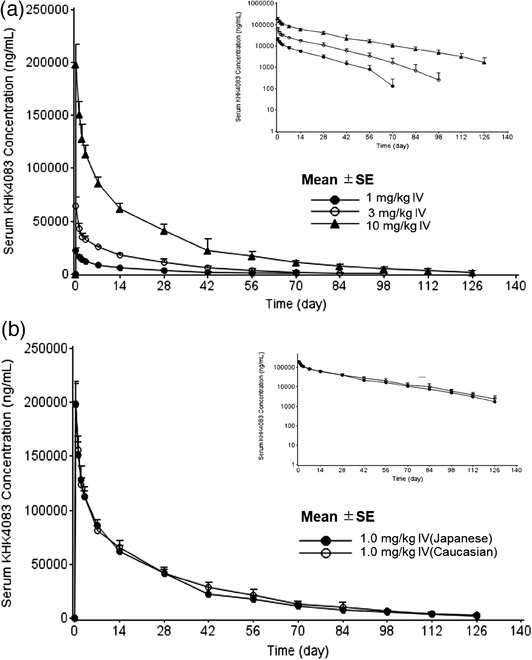
Mean serum concentrations for KHK4083. (a) Single 1, 3, and 10 mg/kg intravenous doses of KHK4083 in healthy Japanese subjects and (b) single 10 mg/kg intravenous dose of KHK4083 in healthy Japanese or Caucasian subjects. *X* axis shows time (day) after the first administration of KHK4083, which was presented as 0 days. Graphs in the upper right corner for (a) and (b): semilog plot of serum concentration. SE, standard error.

Following a single intravenous dose of 1, 3, and 10 mg/kg KHK4083 to healthy Japanese subjects (Figure 2A), C_max_ had increased in a dose‐proportional manner, and AUC_0‐t_, and AUC_0‐∞_ had increased in a more than dose‐proportional manner between 1 and 10 mg/kg (Table 1B). Serum KHK4083 concentration‐time profiles and the mean pharmacokinetic parameters after a single intravenous dose of 10 mg/kg KHK4083 were similar between Japanese and Caucasian healthy subjects (Table 1B and Figure 2B). Following a single subcutaneous administration of KHK4083 at 3 mg/kg, PK exposure (C_max_, AUC_0‐t_, and AUC_0‐∞_) in Japanese and Caucasian healthy subjects were similar (Table 1B). The absolute bioavailability of KHK4083 was estimated to be 58.7% based on the mean AUC_0‐∞_ for subcutaneous and intravenous doses of 3 mg/kg in Japanese subjects.

#### Part 2: Multiple‐Dose Study in UC Patients

Following 3 biweekly intravenous doses of KHK4083 (on days 1, 15, and 29) in patients with UC, C_trough_ (mean ± SD) increased from 64.2 ± 8.9 μg/mL following the dose on day 1 to 124 ± 22 μg/mL after the dose on day 29. C_max_ and AUC_0‐∞_ after the dose in week 4 (day 29) were 297 ± 39 μg/mL and 163 ± 39 mg·h/mL, respectively (Table 1B). Pharmacokinetic parameters for KHK4083 in patients with UC were comparable to those of healthy subjects when we considered the difference in dosing frequency.

### Immunogenicity

#### Part 1: Single‐Ascending‐Dose Study in Healthy Japanese and Caucasian Subjects

In week 0 (predose on day 1), 3 of the 36 subjects (8.3%), 1 subject and 2 subjects administered 3 mg/kg subcutaneous and 10 mg/kg intravenous doses, respectively, were positive for anti‐KHK4083 antibodies (Table 2A). Anti‐KHK4083 antibody positivity showed a tendency to increase over time and to be higher in the subcutaneous group than in the intravenous group. The production of treatment‐boosted ADAs was found in 1 subject (data not shown). Anti‐KHK4083 NAbs were detected in a few subjects.

**Table 2:** Summary of Anti‐KHK4083 Antibody Results: (A) Single Intravenous or Subcutaneous Dose of KHK4083 in Positive Samples of Healthy Japanese or Caucasian Subjects; (B) Intravenous Dose of 10 mg/kg KHK4083 Every 2 Weeks in Positive Samples of Japanese UC Patients

(A) Single Intravenous or Subcutaneous Dose of KHK4083 in Healthy Japanese or Caucasian Subjects–Positive Samples							
		Point					
		Day 1 (Week 0)	Day 29 (Week 4)	Day 71 (Week 10)	Day 99 (Week 14)	Day 127 (Week 18)	End of Treatment
Dose	n	n/n1 (%)	n/n1 (%)	n/n1 (%)	n/n1 (%)	n/n1 (%)	n/n1 (%)
1 mg/kg IV (cohort 1)	6	0/6	0/6	0/6	−	—	—
3 mg/kg IV (cohort 2)	6	0/6	1/6 (16.7)	0/6	1/6 (16.7)	—	—
3 mg/kg SC (cohort 3)	6	0/6	1/6 (16.7)	2/6 (33.3)	4/6 (66.7)	—	—
3 mg/kg SC (Caucasian)							
(cohort 4)	6	1/6 (16.7)	1/6 (16.7)	3/6 (50.0)	3/6 (50.0)	—	—
10 mg/kg IV (cohort 5)	6	0/6	0/6	0/6	0/6	0/6	—
10 mg/kg IV (Caucasian)							
(cohort 6)	6	2/6 (33.3)	3/6 (50.0)	3/6 (50.0)	3/5 (60.0)	3/5 (60.0)	—

(B) Intravenous Dose of 10 mg/kg KHK4083 Q2W in Japanese UC Patients–Positive Samples								
		Point						
		Day 1	Day 29	Day 71	Day 99	Day 127	Day 155	End of
		(Week 0)	(Week 4)	(Week 10)	(Week 14)	(Week 18)	(Week 22)	Treatment
Dose	n	n/n1 (%)	n/n1 (%)	n/n1 (%)	n/n1 (%)	n/n1 (%)	n/n1 (%)	n/n1 (%)
10 mg/kg (UC) (cohort 7)	8	1/8 (12.5)	1/8 (12.5)	0/8	0/8	1/8 (12.5)	2/8 (25.0)	−

IV, intravenous; Q2W, every 2 weeks; SC, subcutaneous; UC, ulcerative colitis; n, number of samples with antidrug antibody‐positive at each time; n1, number of samples at each time.

Percentages are shown in parentheses.

#### Part 2: Multiple‐Dose Study in UC Patients

In week 0 (predose on day 1), 1 of the 8 patients (12.5%) was positive for anti‐KHK4083 antibodies (Table 2B). After the start of the administration of KHK4083, positivity for anti‐KHK4083 antibodies was observed in ≤2 patient at all times. The production of treatment‐boosted ADAs was not observed in any patient. No anti‐KHK4083 NAbs were detected.

### Safety and Tolerability

#### Part 1: Single‐Ascending‐Dose Study in Healthy Japanese and Caucasian Subjects

TEAEs occurred in 23 of 36 subjects (63.9%) in all KHK4083 treatments combined: intravenous dose, 14 of 24 subjects (58.3%); subcutaneous dose, 9 of 12 subjects (75.0%). There were no dose‐related trends in the number of subjects with TEAEs. These TEAEs were reported by 18 of 24 Japanese subjects (75.0%) and 5 of 12 Caucasian subjects (41.7%). In the placebo treatments combined, TEAEs occurred in 5 of 12 subjects (41.7%): Japanese subjects, 4 of 8 (50.0%); Caucasian subjects, 1 of 4 (25.0%).

In KHK4083 treatments combined, drug‐related TEAEs occurred in 21 of 36 subjects (58.3%): intravenous dose, 12 of 24 subjects (50.0%); subcutaneous dose, 9 of 12 subjects (75.0%). These drug‐related TEAEs were reported by 17 of 24 Japanese subjects (70.8%) and 4 of 12 Caucasian subjects (33.3%). In the placebo treatments combined, drug‐related TEAEs occurred in 2 of 12 subjects (16.7%): 1 of 8 Japanese subjects (12.5%) and 1 of 4 Caucasian subjects (25.0%).

The most frequently reported TEAE for subjects in all KHK4083 treatment groups combined was pyrexia, which occurred in 18 of 36 subjects (50.0%): intravenous dose, 10 of 24 subjects (41.7%); subcutaneous dose, 8 of 12 subjects (66.7%). Among the TEAEs reported, all subjects who experienced the events of pyrexia, chills (10 of 36 subjects each), and malaise (7 of 36 subjects) and the high percentage of subjects who experienced the event of headache (9 of 36 subjects) were determined to be drug related. In the placebo group, pyrexia occurred in 2 of 12 subjects (16.7%)—1 subject each in the Japanese and Caucasian cohorts—and the events were considered related to administration (Table 3A).

**Table 3:** Incidence of Drug‐Related TEAEs by SOC and PT: (A) Single‐Ascending‐Dose Study in Healthy Japanese or Caucasian Subjects; (B) Multiple‐Dose Study in Japanese UC Patients

(A) Single‐Ascending‐Dose Study in Healthy Japanese or Caucasian Subjects																
	Placebo				KHK4083											
	Japanese, n = 8		Caucasian, n = 4		1 mg/kg IV, n = 6		3 mg/kg IV, n = 6		3 mg/kg SC, n = 6		3 mg/kg SC (Caucasian), n = 6		10 mg/kg IV, n = 6		10 mg/kg IV (Caucasian), n = 6	
(OSC) PT	n	(%)	n	(%)	n	(%)	n	(%)	n	(%)	n	(%)	n	(%)	n	(%)
Subjects with any drug‐related TEAE	1	(12.5)	1	(25.0)	4	(66.7)	4	(66.7)	6	(100.0)	3	(50.0)	3	(50.0)	1	(16.7)
(Cardiac disorders)	0		0		0		0		1	(16.7)	0		0		0	
Atrioventricular block second degree	0		0		0		0		1	(16.7)	0		0		0	
(Gastrointestinal disorders)	0		0		1	(16.7)	0		0		0		1	(16.7)	0	
Nausea	0		0		1	(16.7)	0		0		0		1	(16.7)	0	
Stomatitis	0		0		0		0		0		0		1	(16.7)	0	
Vomiting	0		0		0		0		0		0		1	(16.7)	0	
(General disorders and administration‐site conditions)	1	(12.5)	1	(25.0)	4	(66.7)	4	(66.7)	5	(83.3)	3	(50.0)	3	(50.0)	0	
Pyrexia	1	(12.5)	1	(25.0)	4	(66.7)	4	(66.7)	5	(83.3)	3	(50.0)	2	(33.3)	0	
Chills	0		0		0		3	(50.0)	2	(33.3)	2	(33.3)	3	(50.0)	0	
Malaise	0		0		2	(33.3)	0		2	(33.3)	1	(16.7)	2	(33.3)	0	
Chest pain	0		0		1	(16.7)	0		0		0		0		0	
(Infections and infestations)	0		0		0		0		0		1	(16.7)	0		0	
Angular cheilitis	0		0		0		0		0		1	(16.7)	0		0	
(Musculoskeletal and connective tissue disorders)	0		0		0		0		1	(16.7)	0		0		0	
Arthralgia	0		0		0		0		1	(16.7)	0		0		0	
(Nervous system disorders)	0		0		0		4	(66.7)	3	(50.0)	1	(16.7)	1	(16.7)	0	
Headache	0		0		0		4	(66.7)	3	(50.0)	1	(16.7)	1	(16.7)	0	
(Renal and urinary disorders)	0		0		1	(16.7)	0		0		0		0		0	
Hematuria	0		0		1	(16.7)	0		0		0		0		0	
(Respiratory, thoracic and mediastinal disorders)	0		0		0		0		0		0		1	(16.7)	0	
Laryngeal inflammation	0		0		0		0		0		0		1	(16.7)	0	
(Skin and subcutaneous tissue disorders)	0		0		0		0		0		0		1	(16.7)	1	(16.7)
Hyperhidrosis	0		0		0		0		0		0		0		1	(16.7)
Seborrheic dermatitis	0		0		0		0		0		0		1	(16.7)	0	

(B) Multiple‐Dose Study in Japanese UC Patients		
	KHK4083	
	10 mg/kg (UC), n = 8	
(OSC) PT	n	%
Subjects with any drug‐related TEAE	5	62.5
(Gastrointestinal disorders)	2	25.0
Stomatitis	2	25.0
Abdominal pain	1	12.5

	KHK4083	
	10 mg/kg (UC), n = 8	
(OSC) PT	n	%
(General disorders and administration‐site conditions)	5	62.5
Chills	4	50.0
Pyrexia	4	50.0
Malaise	1	12.5
(Infections and infestations)	1	12.5
Herpes zoster	1	12.5
(Musculoskeletal and connective tissue disorders)	1	12.5
Arthralgia	1	12.5
(Nervous system disorders)	1	12.5
Headache	1	12.5
(Respiratory, thoracic and mediastinal disorders)	1	12.5
Cough	1	12.5
Oropharyngeal pain	1	12.5

IV, intravenous; PT, preferred term; SC, subcutaneous; OSC, organ system class; TEAEs, treatment‐emergent adverse events.

Coding Dictionary: MedDRA version 20.1.

One of 6 subjects in the 1 and 10 mg/kg intravenous treatment groups and 2 of 8 subjects in the 3 mg/kg subcutaneous treatment group experienced pyrexia of more than 38°C, which showed no dose dependence in its frequency of events. Of 14 subjects in part 1 and part 2 who had experienced chills, 11 subjects also experienced pyrexia (9 of 10 subjects in the KHK4083 treatment group in the single‐ascending‐dose study).

The mean T‐cell counts in the KHK4083 group decreased from the range of 1252.2 to 1752.8/μL at baseline to the range of 608.5 to 842.7/μL on day 2 and recovered to the range of 885.8 to 1373.0/μL in week 1. The mean natural killer (NK) cell counts in the KHK4083 group decreased from the range of 169.7 to 300.5/μL at baseline to the range of 18.2 to 62.8/μL on day 2 and recovered to the range of 93.3 to 180.7/μL in week 1.

#### Part 2: Multiple‐Dose Study in UC Patients

All 8 patients receiving KHK4083 had TEAEs, and of the group, drug‐related TEAEs occurred in 5 patients (62.5%); see Table 3B. The most common TEAEs were chills and pyrexia, which occurred in 4 of 8 patients (50.0%). The next common TEAE was stomatitis, which occurred in 2 patients (25.0%). All these events were considered drug‐related TEAEs. Nasopharyngitis occurred in 2 patients (25.0%) but was considered unrelated to the IP. In part 2, pyrexia occurred in 3 of 8 patients after the first administration and 1 of 8 at 20 days after the last administration. Two of 4 patients who experienced chills also experienced pyrexia.

The mean T‐cell counts decreased from the baseline value of 950.4 to 621.1/μL on day 1 (1 hour after the start of dosing) and recovered to 974.1/μL in week 2 (predose). The mean NK cell counts decreased from 236.0/μL at baseline to 48.5/μL on day 1 (1 hour after the start of dosing) and recovered to 126.4/μL in week 2 (predose).

### Pharmacodynamics

**Figure 3:**
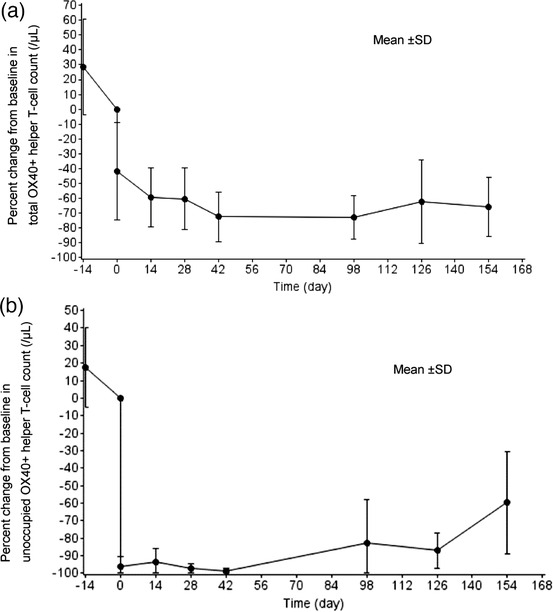
Percent change from baseline for OX40^+^ helper T‐cell count‐time profiles. (a) Total OX40^+^ helper T‐cell count and (b) unoccupied OX40^+^ helper T‐cell count. *X* axis shows time (day) after the first administration. Samples were collected at the screening visit, during the week 0 (predosing and 1 hour after the first dosing) visit, and in the weeks 2, 4, 6, 14, 18, and 22 visits. These sampling points are indicated on the *X* axis as ‐14 days, 0 days (predosing and 1 hour after dosing), and after 14, 28, 42, 98, 126, and 154 days. Percent change was calculated by setting week 0 predosing values as 0 (baseline). OX40^+^, OX40 positive.

The total OX40^+^ Th count (mean ± SD) immediately decreased 1 hour after the first administration on day 1 and remained within the range of ‐59.4% ± 19.8% to ‐72.8% ± 14.6% from day 15 (week 2) to day 155 (week 22); see Figure 3A. The unoccupied OX40^+^ Th count remarkably decreased as far as ‐98.6% ± 1.6% change by day 43 (week 6); see Figure 3B. The unoccupied OX40^+^ Th count recovered to ‐82.6% ± 24.6% by day 99 (week 14).

### Efficacy

The total Mayo score and the partial Mayo score (mean ± SD) decreased from 5.4 ± 1.3 and 4.3 ± 1.8 at baseline to 3.0 ± 1.8 and 2.1 ± 1.5 in week 6, respectively. The partial Mayo score remained persistent after that, scoring 1.9 ± 1.6 in week 22. In week 6, a clinical response was observed in 3 of 8 patients (37.5%), and clinical remission was observed in 2 of 8 patients (25.0%), data not shown.

## Discussion

Overall, KHK4083 administered as a single dose, up to 10 mg/kg intravenously and at 3 mg/kg subcutaneously was generally safe in healthy Japanese and Caucasian subjects. The 10 mg/kg KHK4083 intravenously every 2 weeks was generally safe and well tolerated in patients with moderate to severe UC.

Drug‐related TEAEs occurred in 58.3% of healthy subjects administered KHK4083 and in 62.5% of UC patients. The drug‐related TEAEs were not serious or significant, and did not lead to death or the interruption or discontinuation of IP administration. One Japanese subject who received 3 mg/kg intravenous KHK4083 (cohort 2) and another Japanese subject who received 3 mg/kg subcutaneous KHK4083 (cohort 3) experienced a grade 3 TEAE of pyrexia (related to IP) and enteritis infectious (unrelated to IP), respectively. All other TEAEs were grade 1 or 2 in severity.

The TEAEs that were considered to be associated with acute infusion reactions occurred in more than 50% of healthy subjects or UC patients in the KHK4083 treatment group. Across the KHK4083 single‐intravenous‐dose cohorts, there were no dose‐related trends in the incidence of these events, and they were considered to be related to the IP. A number of subjects experienced pyrexia and chills concurrently (11 of 14 subjects). Acute infusion reactions that include pyrexia and chills are typical symptoms of patients taking monoclonal antibodies.^25^ However, a further investigation should be considered to elucidate the relationship between infusion reactions and the mechanism of action of KHK4083.

No dose‐limiting TEAEs were noted after a single dose of KHK4083 up to 10 mg/kg intravenously or at 3 mg/kg subcutaneously in healthy Japanese and Caucasian subjects and after 10 mg/kg KHK4083 intravenously every 2 weeks in UC patients.

In clinical laboratory tests, almost all subjects experienced transient decreases in T cells and NK cell counts immediately after KHK4083 administration in the single‐ascending‐dose study or immediately after the first administration of KHK4083 in the multiple‐dose study. This decrease in the number of T cells and the number of NK cells was thought to be because of the pharmacological effects of KHK4083, as evident in sources reporting that immunosuppressant activity is observed as a result of the inhibition of OX40.^26^ ^,^ ^27^ No adverse events attributable to such decreases occurred. For other parameters, no clinically significant changes were observed in other test items.

Regarding the mean PK parameters after a single intravenous dose of 1 to 10 mg/kg KHK4083 in healthy Japanese adults, C_max_ showed a dose‐proportional increase. AUC_0‐t_ and AUC_0‐∞_ showed a more than dose‐proportional increase within the range of 1 to 10 mg/kg. The nonlinearity of exposure could imply that KHK4083 exhibited target‐mediated drug disposition, which is typical for monoclonal antibodies.^28^ Serum KHK4083 concentration‐time profiles and mean pharmacokinetic parameters after a single intravenous dose of 10 mg/kg KHK4083 were similar in healthy Japanese and Caucasian subjects. The PK profile in healthy subjects in part 1 and the UC patients in part 2 were similar. The consistency observed in the PK profiles in subject groups with different demographic characteristics showed that baseline characteristics are not likely to affect the exposure of KHK4083 in subjects receiving the drug.

Results of ADA production showed an increase in percentage over time. In part 2, 1 of the 8 subjects was positive for anti‐KHK4083 antibodies at baseline. However, studies show that the presence of ADA at baseline is common for biological drugs.^29^ Moreover, immunogenicity rate variations are strongly reflected by differences in the assays used to measure the ADAs.^30^ The absence of NAbs also shows that the expression of antibodies is likely to be for the above reasons.

In part 2, the total OX40^+^ Th decreased immediately after the initiation of treatment with KHK4083 and unoccupied OX40^+^ Th counts, which remarkably decreased immediately after the initiation of day 1 KHK4083 administration (1 hour after day 1 IP administration), reaching below 5% of the baseline value. Our results for the pharmacodynamic assessment showed that the pharmacological action of KHK4083 depleted OX40‐expressing cells; this effect was persistent, suggesting that KHK4083 may play a role as a new target‐specific, selective drug for UC.

The exploratory analyses of clinical efficacy showed improvements in Mayo scores. The total Mayo score and the partial Mayo score decreased in week 6, and the partial Mayo score remained constant until week 22. The clinical response and clinical remission achieved by the FAS in week 22 were 37.5% and 25.0%, respectively, suggesting efficacious implications of KHK4083 in patients with moderate to severe UC.

Limitations of this study included the small study size—the small number of patients analyzed—and the lack of a control group for the efficacy analysis in part 2, which might not have allowed an appropriate evaluation reflecting the population. In addition, the current data were not adjusted for possible confounders, including demographic characteristics. Hence, the reliability of clinical assessment data could be enhanced in further studies.

In conclusion, our study demonstrated the safety and tolerability of single intravenous or subcutaneous administration of KHK4083 in healthy Japanese and Caucasian subjects, as well as the safety and tolerability of multiple doses of KHK4083 in patients with moderate to severe UC, by comparison with the results obtained by healthy subjects. Moreover, pharmacological discrepancies were not observed between the profiles of Japanese and Caucasian subjects. Favorable pharmacological actions were evident from the results with the OX40+ cells and improvements in the symptoms of UC patients. These clinical data suggest considerations for further study on evaluating the efficacy of KHK4083 in patients with severe UC.

## Conflicts of Interest

Norihito Watanabe, MD (Division of Gastroenterology, Tokai University Hachioji Hospital, Tokyo, Japan) received consultant fees, research grants, and speaker honoraria from Kyowa Kirin Co., Ltd. Kenichi Furihata, Yoh Ishiguro, Naoki Yoshimura, Hiroaki Ito, and Shinji Katsushima are the investigators of this study and declare no conflicts of interest. Etsuji Kaneko, Munetake Shimabe, Mayumi Mukai, Risa Watanabe, and Takuya Morishige are employees of Kyowa Kirin Co., Ltd.

## Funding

This work was funded and supported by Kyowa Kirin Co., Ltd.

## Supporting information

Supporting Information

Supporting Information

Supporting Information

## References

[1] Papp KA, Gooderham MJ, Girard G, Raman M, Strout V. Phase I randomized study of KHK4083, an anti‐OX40 monoclonal antibody, in patients with mild to moderate plaque psoriasis. J Eur Acad Dermatol Venereol. 2017;31(8):1324‐1332. 28556418 10.1111/jdv.14313 PMC5575535

[2] Croft M. The role of TNF superfamily members in T‐cell function and diseases. Nat Rev Immunol. 2009;9(4):271‐285. 19319144 10.1038/nri2526 PMC2737409

[3] Croft M. Control of immunity by the TNFR‐related molecule OX40 (CD134). Annu Rev Immunol. 2010;28:57‐78. 20307208 10.1146/annurev-immunol-030409-101243 PMC2882161

[4] Gao J, Bernatchez C, Sharma P, Radvanyi LG, Hwu P. Advances in the development of cancer immunotherapies. Trends Immunol. 2013;34(2):90‐98. 23031830 10.1016/j.it.2012.08.004 PMC3565019

[5] Nakagawa H, Iizuka H, Nemoto O, et al. Safety, tolerability and efficacy of repeated intravenous infusions of KHK4083, a fully human anti‐OX40 monoclonal antibody, in Japanese patients with moderate to severe atopic dermatitis. J Dermatol Sci. 2020;99(2):82‐89. 32651105 10.1016/j.jdermsci.2020.06.005

[6] Abraham C, Medzhitov R. Interactions between the host innate immune system and microbes in inflammatory bowel disease. Gastroenterology. 2011;140(6):1729‐1737. 21530739 10.1053/j.gastro.2011.02.012 PMC4007055

[7] El‐Hodhod MA, Aly RH, Youssef SR, Mohamed SI. Enhanced blood lymphocytes apoptosis in children with inflammatory bowel disease. ISRN Gastroenterol. August 29, 2013;2013:415417. 24073337 10.1155/2013/415417 PMC3773421

[8] Ungaro R, Mehandru S, Allen PB, Peyrin‐Biroulet L, Colombel JF. Ulcerative colitis. Lancet. 2017;389(10080):1756‐1770. 27914657 10.1016/S0140-6736(16)32126-2 PMC6487890

[9] Kmieć Z, Cyman M, Ślebioda TJ. Cell of the innate and adaptive immunity and their interactions in inflammatory bowel disease. Adv Med Sci. 2017;62(1):1‐16. 28126697 10.1016/j.advms.2016.09.001

[10] Khor B, Gardet A, Xavier RJ. Genetics and pathogenesis of inflammatory bowel disease. Nature. 2011;474(7351):307‐317. 21677747 10.1038/nature10209 PMC3204665

[11] Mahmood T, Yang PC. OX40L‐OX40 interactions: a possible target for gastrointestinal autoimmune diseases. *N* Am J Med Sci. 2012;4(11):533‐536. 10.4103/1947-2714.103311 PMC3503370 23181223

[12] Kita T, Kaji Y, Kitamura K. Safety, tolerability, and pharmacokinetics of adrenomedullin in healthy males: a randomized, double‐blind, phase 1 clinical trial. Drug Des Devel Ther. January 6, 2020:14:1‐11. 10.2147/DDDT.S225220 PMC6955635 32021087

[13] Molodecky NA, Soon IS, Rabi DM, et al. Increasing Incidence and prevalence of the inflammatory bowel diseases with time, based on systematic review. Gastroenterology. 2012;142(1):46‐54.e42. 22001864 10.1053/j.gastro.2011.10.001

[14] Sifers T, Hirten R, Mehandru S, Ko HM, Colombel JF, Cunningham‐Rundles C. Vedolizumab therapy in common variable immune deficiency associated enteropathy: A case series. Clin Immunol. March, 2020;212:108362. 32058070 10.1016/j.clim.2020.108362 PMC7310569

[15] Rubin DT, Ananthakrishnan AN, Siegel CA, et al. ACG clinical guideline: ulcerative colitis in adults. Am J Gastroenterol. 2019;114(3):384‐413. 30840605 10.14309/ajg.0000000000000152

[16] Stüber E, Büschenfeld A, Lüttges J, Von Freier A, Arendt T, Fölsch UR. The expression of OX40 in immunologically mediated diseases of the gastrointestinal tract (celiac disease, Crohn's disease, ulcerative colitis). Eur J Clin Invest. 2000;30(7):594‐599. 10886299 10.1046/j.1365-2362.2000.00658.x

[17] Souza HS, Elia CC, Spencer J, Macdonald TT. Expression of lymphocyte‐endothelial receptor‐ligand pairs, alpha4beta7/MAdCAM‐1 and OX40/OX40 ligand in the colon and jejunum of patients with inflammatory bowel disease. Gut. 1999;45(6):856‐863. 10562584 10.1136/gut.45.6.856 PMC1727744

[18] Zhao Z, Gu Y, Miao D, Hoffmeyer E, Liu Y, Yu L. Determination of autoantibodies to transglutaminase by electrochemiluminescence (ECL) assay. Methods Mol Biol. 2019;1901:197‐203. 30539579 10.1007/978-1-4939-8949-2_16

[19] Wu Y, Liu X, Chen Y, Woods R, Lee N, Yang H, Chowdhury P, Roskos LK, White WI, An electrochemiluminescence (ECL)‐based assay for the specific detection of anti‐drug antibodies of the IgE isotype. J Pharm Biomed Anal. December, 2013;86:73‐81. 23988731 10.1016/j.jpba.2013.06.005

[20] World Health Organization. Laboratory Guidelines for enumerating CD4 T Lymphocytes in the context of HIV/AIDS. New Delhi, India: Regional Office for Sounth‐East Asia; 2009.

[21] Liang M, Schwickart M, Schneider AK, et al. Receptor occupancy assessment by flow cytometry as a pharmacodynamic biomarker in biopharmaceutical development. Cytometry B Clin Cytom. 2016;90(2):117‐127. 26054054 10.1002/cyto.b.21259 PMC5042057

[22] Food and Drug Administration Center for Drug Evaluation and Research. Ulcerative Colitis: Clinical Trial Endpoints Guidance for Industry DRAFT GUIDANCE. Washington, DC: U.S. Department of Health and Human Services; 2016.

[23] Higgins P D R, Schwartz M, Mapili J, Krokos I, Leung J, Zimmermann EM. Patient defined dichotomous end points for remission and clinical improvement in ulcerative colitis. Gut. 2005;54(6):782‐788. 15888785 10.1136/gut.2004.056358 PMC1774553

[24] Lewis JD, Chuai S, Nessel L, Lichtenstein GR, Aberra FN, Ellenberg JH. Use of the noninvasive components of the Mayo score to assess clinical response in ulcerative colitis. Inflamm Bowel Dis. 2008;14(12):1660‐1666. 18623174 10.1002/ibd.20520 PMC2597552

[25] Dillman RO. Infusion reactions associated with the therapeutic use of monoclonal antibodies in the treatment of malignancy. Cancer Metastasis Rev. 1999;18(4):465‐471. 10855789 10.1023/a:1006341717398

[26] Boleto G, Allanore Y, Avouac J. Targeting costimulatory pathways in systemic sclerosis. Front Immunol. December, 2018;9:2998. 30619351 10.3389/fimmu.2018.02998 PMC6305435

[27] Webb GJ, Hirschfield GM, Lane PJ. OX40, OX40L and autoimmunity: a comprehensive review. Clin Rev Allergy Immunol. 2016;50(3):312‐332. 26215166 10.1007/s12016-015-8498-3

[28] Cao Y, Jusko WJ. Incorporating target‐mediated drug disposition in a minimal physiologically‐based pharmacokinetic model for monoclonal antibodies. J Pharmacokinet Pharmacodyn. 2014;41(4):375‐387. 25077917 10.1007/s10928-014-9372-2 PMC4167346

[29] Gorovits B, Clements‐Egan A, Birchler M, et al. Pre‐existing antibody: biotherapeutic modality‐based review. AAPS J. 2016;18(2):311‐320. 26821802 10.1208/s12248-016-9878-1 PMC4779092

[30] Gorovits B, Baltrukonis DJ, Bhattacharya I, et al. Immunoassay methods used in clinical studies for the detection of anti‐drug antibodies to adalimumab and infliximab. Clin Exp Immunol. 2018;192(3):348‐365. 29431871 10.1111/cei.13112 PMC5980437

