# Supplementary material for: A Phase 1 Study of KHK4083: A Single‐Blind, Randomized, Placebo‐Controlled Single‐Ascending‐Dose Study in Healthy Adults and an Open‐Label Multiple‐Dose Study in Patients With Ulcerative Colitis
Source: Clin Pharmacol Drug Dev. 2021 Jan 29;10(8):870–83. doi: 10.1002/cpdd.918 (PMC8451804; doi:10.1002/cpdd.918)
Supplement: Supplementary file 2 — Supporting Information [file CPDD-10-870-s002.pdf]

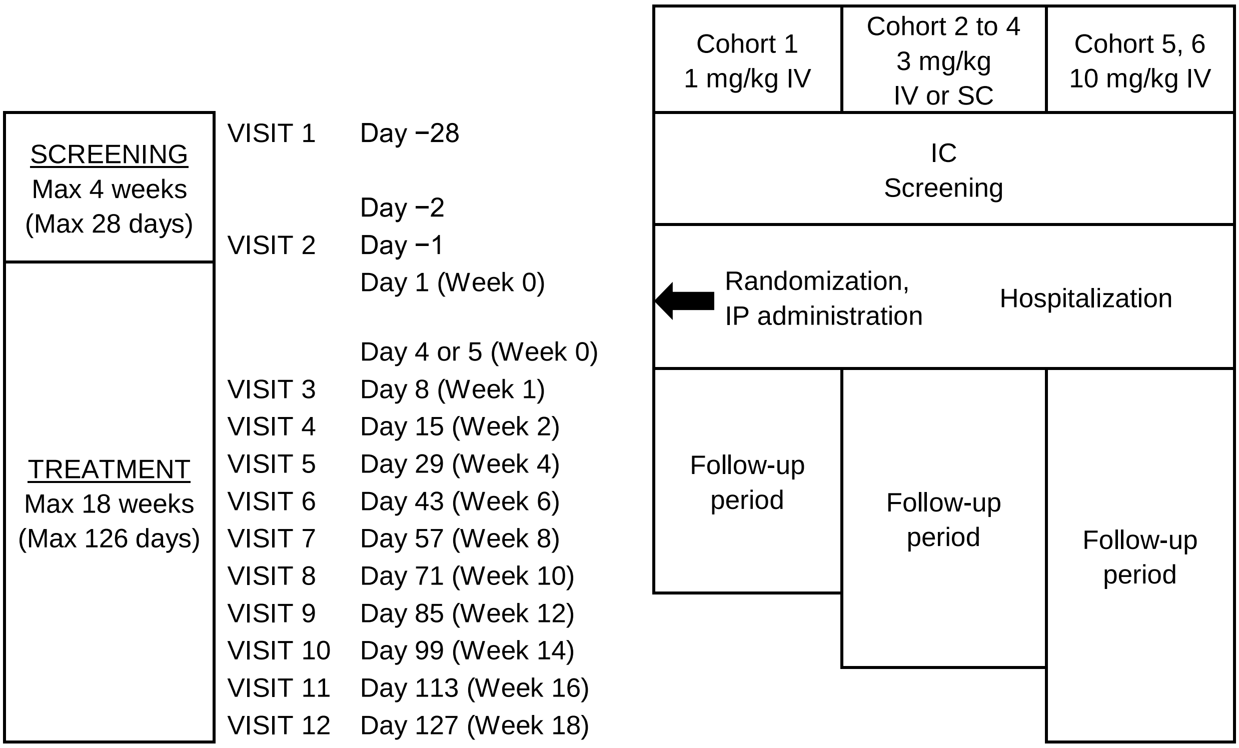

SCREENING
Max 4 weeks
(Max 28 days)
TREATMENT
Max 18 weeks
(Max 126 days)
VISIT 1 Day −28
Day −2
VISIT 2 Day −1
Day 1 (Week 0)
Day 4 or 5 (Week 0)
VISIT 3 Day 8 (Week 1)
VISIT 4 Day 15 (Week 2)
VISIT 5 Day 29 (Week 4)
VISIT 6 Day 43 (Week 6)
VISIT 7 Day 57 (Week 8)
VISIT 8 Day 71 (Week 10)
VISIT 9 Day 85 (Week 12)
VISIT 10 Day 99 (Week 14)
VISIT 11 Day 113 (Week 16)
VISIT 12 Day 127 (Week 18)
Cohort 1
1 mg/kg IV
Cohort 2 to 4
3 mg/kg
IV or SC
Cohort 5, 6
10 mg/kg IV
IC
Screening
Randomization,
IP administration
Hospitalization
Follow-up period
Follow-up period
Follow-up period
