# Supplementary material for: A Phase 1 Study of KHK4083: A Single‐Blind, Randomized, Placebo‐Controlled Single‐Ascending‐Dose Study in Healthy Adults and an Open‐Label Multiple‐Dose Study in Patients With Ulcerative Colitis
Source: Clin Pharmacol Drug Dev. 2021 Jan 29;10(8):870–83. doi: 10.1002/cpdd.918 (PMC8451804; doi:10.1002/cpdd.918)
Supplement: Supplementary file 3 — Supporting Information [file CPDD-10-870-s003.pdf]

Period	Visit	Day	Cohort 7<br>UC patients<br>($N \geq 4$)
SCREENING<br>Max 4 weeks<br>(Max 28 days)	VISIT 1	Day −28<br><br>Day −1	IC<br>Screening<br>Enrollment
TREATMENT<br>22 weeks<br>(155 days)	VISIT 2	Day 1 (Week 0)	← KHK4083 multiple IV administration. Dosage determined by safety assessments from Cohort 5 or 6.
	VISIT 3	Day 15 (Week 2)	←
	VISIT 4	Day 29 (Week 4)	←
	VISIT 5	Day 43 (Week 6)	Follow-up period
	VISIT 6	Day 71 (Week 10)	
	VISIT 7	Day 99 (Week 14)	
	VISIT 8	Day 127 (Week 18)	
	VISIT 9	Day 155（Week 22）	
